# An Interpretable Machine Learning Approach to Ecologically Characterize Soil Carbon and Structure From Multi‐Kingdom Microbiome, Texture and Climate

**DOI:** 10.1111/mec.70535

**Published:** 2026-09-01

**Authors:** Thomas Jeanne, Julien Prunier, Richard Hogue, Arnaud Droit

**Affiliations:** ^1^ Computational Biology Laboratory Centre de Recherche du CHU de Québec—Université Laval Quebec Canada; ^2^ Institut de Recherche et de Développement en Agroenvironnement Quebec Canada; ^3^ Genovalia, Centre de Valorisation de données génomiques Non‐Humaines Université Laval Quebec Canada

**Keywords:** interpretable machine learning, soil carbon stock, soil microbiome, soil structure

## Abstract

Soil physical structure is a critical determinant of agricultural landscape resilience, yet standard pedotransfer functions estimate soil hydraulic and structural properties using static abiotic variables, often overlooking the biological mechanisms that actively organize soil structure. This study evaluates the predictive power of multi‐kingdom microbiome data (prokaryotes, fungi and microeukaryotes) for three key soil functions: soil organic carbon (SOC) stock, mean weight diameter (MWD) and macroporosity. Using a dataset of 2251 agricultural soil samples from Quebec, Canada, we benchmarked four machine learning algorithms (HGBR, RFR, XGBoost, SVR) and four data aggregation strategies. The integration of microbiome data with texture and climate variables achieved high peak predictive accuracy ($R^{2}$ range: 0.70–0.82). Methodologically, high‐resolution compositional approaches (ASV‐level centered log‐ratio) and kingdom‐balanced absolute abundances consistently outperformed taxonomic or functional aggregations. The loss of predictive power at the family level indicates that traits governing soil physical modification are phylogenetically shallow and strain‐specific. Interpretability analysis using Shapley Additive Explanations (SHAP) revealed a clear functional hierarchy in soil assembly. Specific prokaryotic and fungal features drove biochemical stabilization and physical scaffolding via the microbial carbon pump and structural enmeshment dynamics. In contrast, the architectural openness of macroporosity was fundamentally constrained by abiotic physical limits (e.g., texture). Within this physical framework, specific microbial taxa, including anaerobic bacteria and microeukaryotic amoebae, functioned not as active engineers, but as high‐sensitivity bio‐indicators of the resulting aeration and hydrological connectivity. These results define soil physical organization as a biologically mediated hierarchy rather than a passive geological byproduct. Consequently, we propose shifting from static pedotransfer functions to a dynamic biotransfer framework that leverages multi‐kingdom omic signatures to monitor soil physical resilience and crop adaptation potential.

## 1 Introduction

### 1.1 The Soil Microbiome as a Biophysical Driver of Crop Adaptation

Understanding the mechanisms that facilitate crop adaptation to changing climates is a central goal of agricultural landscape genomics. Although much attention focuses on plant genomic variation, crop resilience is equally dependent on the extended genome of the soil microbiome, particularly its role in maintaining the physical environment. This study addresses the specific topic of ‘Microbiome impacts on crop adaptation’ by elucidating how microbial communities engineer the soil structure necessary for root proliferation and water resilience. Soil health, though a complex concept, is fundamentally based on the feedback loops between organic matter processes and physical structure (Kibblewhite et al. 2008). Soil organic carbon (SOC) serves as the primary proxy for fertility and energy storage, governing nutrient availability, water retention, biological activity and climate regulation (Lal 2016; Amundson et al. 2015; Minasny et al. 2017). While SOC turnover is essential for plant growth and soil stability, recent models emphasize that persistence is primarily governed by environmental constraints, physical accessibility and occlusion (Lehmann and Kleber 2015; Schmidt et al. 2011; Dungait et al. 2012). Concurrently, soil structure, characterized by properties such as aggregate stability, defines the energetic and physical stability of the soil matrix. Following recent literature, it is essential to distinguish this from soil architecture (Yudina and Kuzyakov 2023). Whereas structure encompasses the chemical and energetic binding of aggregates, architecture strictly refers to the geometric and topological arrangement of the pore‐solid interface. Therefore, it is this architecture, including macroporosity and pore connectivity, that defines the physical habitat for soil organisms and the path of least resistance for plant roots. Soil aggregates act as physical barriers that shield organic matter from enzymatic degradation while providing protected micro‐habitats for microbial colonies, sheltering them from predation and environmental fluctuations (Six et al. 2004; Tisdall and Oades 1982). Well‐formed soils with sufficient macroporosity facilitate water infiltration, gas exchange and root penetration, simultaneously ensuring the spatial connectivity required for the diffusion of nutrients and the active movement of microorganisms toward nutrient‐rich hotspots (Rabot et al. 2018). Integrating these three properties provides a comprehensive view of how structural degradation affects carbon storage and hydrological dynamics.

### 1.2 The Role of the Microbiome, Soil Texture and Climate as Predictors

Soil functionality results from complex interactions between plants and microorganisms, as well as competitive and synergistic relationships within and across microbial kingdoms. These processes occur across multiple spatial and temporal scales, requiring a predictive framework that integrates inherent physical properties with dynamic biological agents. Traditionally, pedotransfer functions have modelled soil properties based on texture and bulk density (which define static physical limits) and climatic variables (which influence thermodynamic rates) (McBratney et al. 2003; Sinsabaugh et al. 2008). While these abiotic factors act as distal drivers, they often fail to capture the small‐scale heterogeneity driven by proximate biological activity.

Soil microorganisms, including prokaryotes, fungi and microeukaryotes, act as the primary engineers of the soil environment. Beyond inhabiting the physical matrix described previously, they drive nutrient mineralization, produce the biopolymers and hyphal networks necessary for macro‐aggregate formation and regulate organic matter decomposition (Wagg et al. 2014; Delgado‐Baquerizo et al. 2016). For instance, bacterial growth, mortality and necromass production significantly influence organic carbon persistence (Liang et al. 2017). This relationship is also reciprocal, as microorganisms are shaped by their environment while simultaneously modifying it (Philippot et al. 2024). This integrative capacity establishes the soil microbiome as a sensitive biosensor of environmental history and management practices.

Therefore, supplementing standard pedoclimatic data with microbiome profiles, particularly those obtained through amplicon sequencing, provides a critical mechanistic layer for soil characterization. This holistic approach enables the identification of higher‐resolution predictors, capturing the biological potential that is inherently overlooked in purely physical models (Fierer 2017; Bastida et al. 2021).

### 1.3 Machine Learning and Predictive Modelling in Soil Science

The combination of high‐throughput sequencing with pedoclimatic datasets provides a strong path for predictive soil ecology, but it also presents specific analytical challenges. Microbiome data are inherently sparse, compositional and high‐dimensional, which breaches the assumptions of normality and independence needed in most parametric statistics (Gloor et al. 2017). Additionally, the relationships between microbial communities and soil functions are likely driven by highly non‐linear dynamics and complex conditional dependencies.

To address these complexities, supervised machine learning (ML) algorithms have emerged as powerful tools. Benchmarking studies demonstrate that tree‐based ensemble methods, such as Random Forest Regression (RFR), eXtreme Gradient Boosting (XGBoost) and Histogram Gradient Boosting Regression (HGBR), alongside kernel‐based models like Support Vector Regression (SVR), are especially effective at capturing the high‐dimensional structure of microbial data (Topçuoğlu et al. 2020; Thompson et al. 2019). These algorithms robustly handle multicollinearity and sparsity without requiring a priori mechanistic definitions.

However, model efficacy heavily depends on feature preprocessing. Strategies that effectively reduce dimensionality and thus likely enhance generalization (Knight et al. 2018) often combine amplicon sequence variants (ASVs) into broader taxonomic groups or infer functional profiles. Yet, this aggregation involves a critical trade‐off, risking the loss of fine‐scale phylogenetic signals or strain‐specific traits that may govern specific soil functions (Martiny et al. 2015). Furthermore, a truly comprehensive framework must incorporate prokaryotic, fungal and microeukaryotic datasets. Merging these distinct amplicon libraries presents unique challenges related to extraction efficiencies, ribosomal gene copy numbers and sequencing depths (McLaren et al. 2019; Větrovský and Baldrian 2013). To extract actionable knowledge and build trust in these complex models, model‐agnostic interpretation tools like Shapley Additive Explanations (SHAP) have proven highly effective (Lundberg and Lee 2017). Despite these advances in model explainability, systematic evaluations of how data integration and aggregation strategies affect model performance remain limited, especially concerning multi‐trophic microbial guilds (Wilhelm et al. 2022).

This study addresses these gaps by developing ML models to predict key soil biophysical properties (SOC stock, mean weight diameter and macroporosity) using multi‐kingdom microbiome profiles alongside standard soil texture and climate data. Specifically, we aimed to: (i) systematically evaluate model performance across four distinct microbiome data aggregation strategies and (ii) employ SHAP values to extract detailed ecological interpretations of the most influential predictors. Ultimately, this approach seeks to simultaneously maximize the predictive accuracy and the ecological explainability of machine learning in soil science.

## 2 Materials and Methods

### 2.1 Study Context and Data Provenance

**FIGURE 1:**
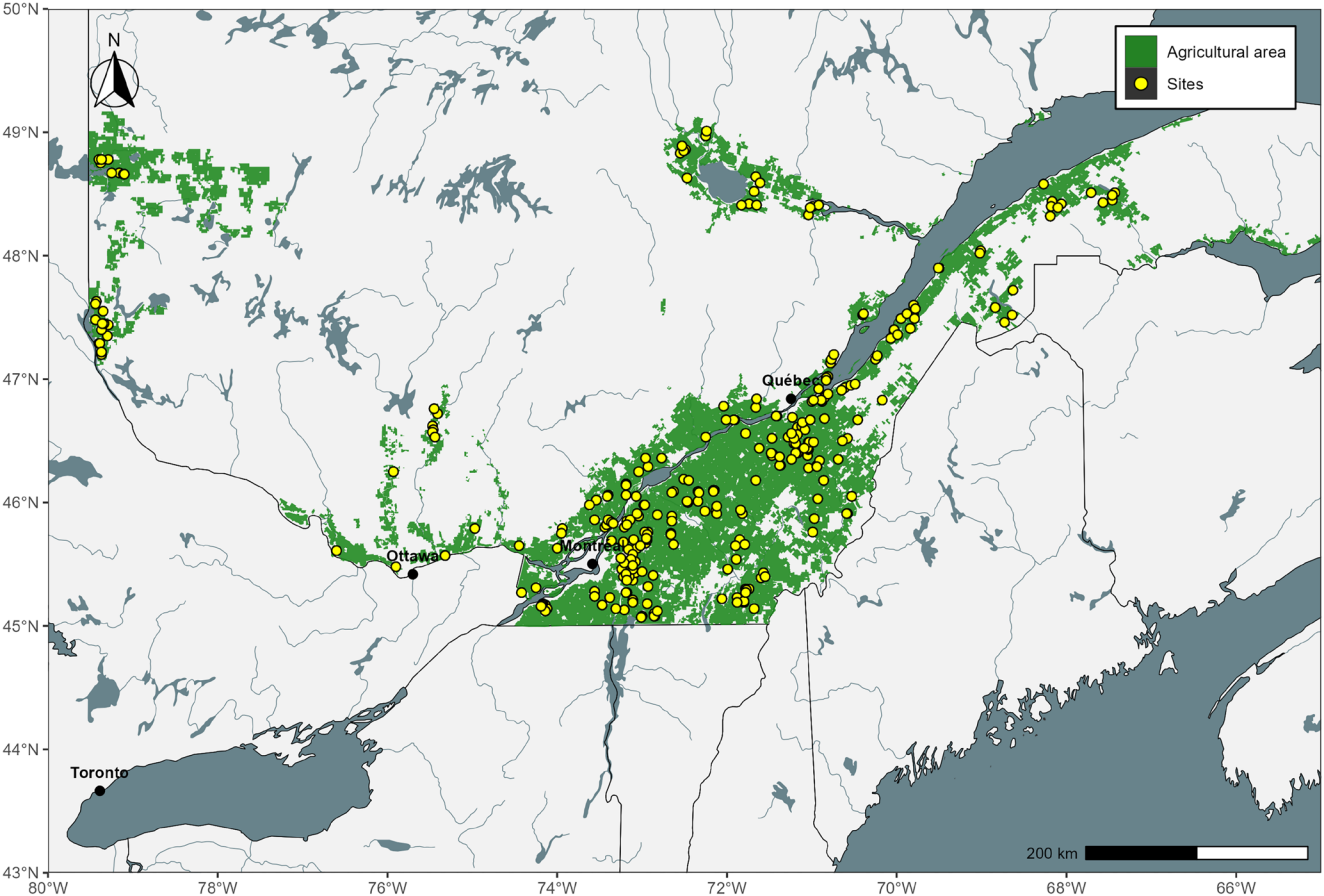
Distribution of soil sampling sites in Quebec within the provincial agricultural area. The map displays the spatial distribution of soil microbiome sampling sites across Quebec, overlaid with the official agricultural area boundaries (Zone agricole transposée au Cadastre du Québec (Commission de protection du territoire agricole 2025)).

The data used in this study were obtained from the large‐scale Quebec Soil Health Assessment (EESSAQ) project. While the overarching project was conducted from 2017 to 2025 in collaboration with the Quebec Ministry of Agriculture, Fisheries and Food (Ministère de l'Agriculture, des Pêcheries et de l'Alimentation du Québec; MAPAQ), the specific soil samples analyzed here (N=2,251) were collected during the 2018 and 2019 agricultural campaigns from 303 agricultural fields (Figure 1), covering 71 different soil series across Quebec (Gasser et al. 2023; Jeanne et al. 2025). Each field was sampled at four different points, typically spaced 50 m apart. At each sampling point, the agricultural surface horizon (Ap) and the underlying B horizon were identified based on standard pedological criteria (Soil Classification Working Group 1998). When visible structural differences were present within the surface layer, the Ap horizon was further subdivided and sampled as distinct Ap1 and Ap2 sub‐horizons. In cases where the Ap horizon was relatively shallow (e.g., 15–20 cm), a single Ap sample was collected. The underlying B horizon was consistently sampled across all sites. All samples were properly stabilized according to the requirements of the planned analysis.

Although sampled exclusively within Quebec, this dataset encompasses a broad pedoclimatic gradient, ranging from sandy to skeletal, loamy, clayey and glacial soils. Because the models capture fundamental abiotic constraints (texture and climate) alongside the microbiome, the predictive frameworks developed here have the potential to be extrapolated to other agricultural regions with similar soil and climatic characteristics.

### 2.2 Input Variables: Microbiome, Soil Texture and Climate

The input variables for the machine learning models consisted of three main categories:

#### 2.2.1 Microbiome Data

Soil DNA extraction, followed by molecular analysis, processing and filtering, was performed as previously described (Jeanne et al. 2025). In brief, DNA from soil samples in the Ap horizons was extracted using the FastDNA SPIN Kit for Soil (MP Biomedicals, Solon, OH, USA). Amplicon sequencing targeted the 16S rRNA gene for prokaryotes (Apprill et al. 2015; Parada et al. 2016), the ITS1 region for fungi (Bokulich and Mills 2013) and the 18S rRNA gene for microeukaryotes (Comeau et al. 2011). Sequencing was carried out on Illumina instruments by the Plateforme d'analyses génomiques (IBIS, Université Laval, Québec City, QC, Canada). Raw sequence data were processed through a standardized bioinformatics pipeline in QIIME2 version 2023.9 (Bolyen et al. 2019) to produce amplicon sequence variant (ASV) tables and taxonomic assignments for each microbial group. The taxonomic database UNITE version 9 (Nilsson et al. 2019) was used for fungi, while SILVA version 138 (Quast et al. 2012) was used for prokaryotes and microeukaryotes.

#### 2.2.2 Soil Texture

The characterization of soil texture in this study was performed using the sedimentation method, following the detailed procedures outlined by Gee and Bauder (1986). This technique is based on Stokes' Law, which relates the settling velocity of particles in a fluid to their size, assuming spherical particles and a constant temperature. The final result for each soil sample was expressed as the percentage by weight of sand, silt and clay, which collectively define the soil's textural class using the USDA soil textural triangle.

#### 2.2.3 Climate Data

Daily climatic variables, including precipitation and temperature, were obtained using Daymet version 4 (Thornton et al. 2021). Rather than using a 30‐year climate normal, daily climatic data for each site were collected specifically over the 5 years strictly prior to its respective sampling date (2018/2019). This medium‐term window was selected to capture the recent climatic conditions most directly responsible for shaping the current microbial community structures. Total precipitation (PPT), growing degree days (GDD) and the Gini index (Sahbeni et al. 2023) were calculated for the average growing season in the province of Quebec (April 1 to October 31).

It is important to note that variables related to agricultural management (e.g., tillage, cover crops, crop rotation) were deliberately excluded from these predictive models. Our objective was to evaluate the baseline predictive capacity of the microbiome coupled strictly with inherent abiotic factors (climate and texture), reserving management variables for future integration into decision‐support tools. Furthermore, preliminary modelling indicated that adding physicochemical variables such as soil pH did not improve model performance. This strongly suggests that the significant influence of pH on soil biology is already inherently captured within the microbial community composition data.

### 2.3 Output Variables: Soil Carbon Stock, Soil Aggregate Size and Stability, Macroporosity

Our predictive models were developed to forecast three key structural and functional soil properties: soil organic carbon (SOC) stock, mean weight diameter (MWD) as a proxy for aggregate stability and macroporosity.

#### 2.3.1 Soil Organic Carbon (SOC) Stock

The SOC stock was measured and expressed as megagrams of carbon per hectare (Mg C ha^−1^). For each soil layer, carbon stock calculations were performed using bulk density measurements. Bulk density was determined using the core method, which involves collecting intact soil cores of a known volume, drying them at 105°C until reaching a constant weight and calculating mass per volume. The C content of each soil layer was measured after sieving dried soils at 100 mesh and analysing them by dry combustion (LECO CN828, LECO Corporation, St. Joseph, MI, USA) (Nelson and Sommers 1996). Because agricultural soils in Quebec are naturally poor in inorganic carbonates, and internal laboratory validations confirmed the absence of carbonates in samples with a pH <7.2 (with only 3.9% of the study samples exhibiting a pH >7.2), the measured total carbon was considered directly equivalent to the soil organic carbon (SOC). Therefore, no prior acid treatment was required to eliminate inorganic carbon. SOC stock was then calculated for each layer and summed across the profile as described in Equation (1):

(1) $${\mathrm{SOC}}_{\text{stock}}=\sum_{i=1}^{n}C_{i}\times {\mathrm{BD}}_{i}\times T_{i}\times \left(1-\theta_{i}\right)\times 0.1$$

where ${\mathrm{SOC}}_{\text{stock}}$ is the soil organic carbon stock (Mg C ha^−1^); $C_{i}$ is the organic carbon concentration in layer i (g C kg^−1^); ${\mathrm{BD}}_{i}$ is the bulk density (g cm^−3^); $T_{i}$ is the layer thickness (cm); $\theta_{i}$ is the volume fraction of coarse fragments (dimensionless); the factor 0.1 converts units to Mg C ha^−1^; and n is the number of soil layers.

#### 2.3.2 Mean Weight Diameter (MWD)

The soil aggregate size and stability were assessed in the Ap horizon and expressed as the mean weight diameter (MWD) in millimetres (mm). A standardized wet‐sieving procedure, following the protocol outlined by Angers and Mehuys (1993), was employed to determine MWD. Briefly, an initial mass of 50 g of fresh soil aggregates was placed on the top sieve of a nest of sieves (mesh sizes: 8 mm, 4 mm, 2 mm, 1 mm, 0.5 mm and 0.25 mm). Concurrently, a separate subsample of the fresh soil was oven‐dried to determine its gravimetric water content, allowing the exact equivalent dry mass of the initial 50 g sample to be calculated. The rainfall impact and slaking effect were simulated by subjecting the sieves to 10 min of vertical oscillation in water (3.5 cm stroke length at 30 strokes/min). After sieving, the soil retained on each individual sieve was carefully collected, oven‐dried at 105°C until a constant weight was achieved, and then weighed. Furthermore, the mass of soil retained on the sieves was corrected for sand content by dispersing the aggregates with sodium hexametaphosphate and subtracting the oven‐dried weight of the retained sand fraction. The MWD was subsequently calculated using the corrected mass of soil retained on each sieve and the corresponding sieve mesh size, as defined in Equation (2):

(2) $$\mathrm{MWD}=\sum_{i=1}^{n}x_{i}\times w_{i}$$

where MWD is the mean weight diameter of soil aggregates (mm); $x_{i}$ is the mean diameter of aggregates in size class i (mm); $w_{i}$ is the proportion of the total dry mass in size class i (dimensionless); and n is the number of aggregate size classes.

#### 2.3.3 Macroporosity

Macroporosity determination involved collecting intact soil cores in the field and transporting them undisturbed to the laboratory. The cores were saturated with water, and total porosity was defined as the volumetric water content at saturation. The cores were then equilibrated at a matric tension of 10 kPa using a tension plate. In this study, macroporosity is functionally defined as the pore volume with an equivalent diameter > 30 μm, which corresponds to the volume of water drained between saturation and 10 kPa tension (Reynolds et al. 2009; Carter and Gregorich 2008). After equilibration, the cores were oven‐dried at 105°C to a constant mass to determine the dry soil bulk density. Macroporosity (cm^3^ cm^−3^) was calculated as the difference between the volumetric water content at saturation and at 10 kPa. To obtain a representative value for the entire soil profile, the resulting macroporosity was computed as a thickness‐weighted average, as defined in Equation (3):

(3) $$P_{\text{macro},\text{profile}}=\frac{\sum_{i=1}^{n}P_{\text{macro},i}\times T_{i}}{\sum_{i=1}^{n}T_{i}}$$

where $P_{\text{macro},\text{profile}}$ is the profile‐level macroporosity (cm^3^ cm^−3^); $P_{\text{macro},i}$ is the macroporosity of soil layer i (cm^3^ cm^−3^); $T_{i}$ is the thickness of layer i (cm); $\sum_{i=1}^{n}T_{i}$ is the total profile thickness (cm); and n is the number of soil layers.

While output variables such as SOC stock and macroporosity were calculated for the entire soil profile, the microbiome predictors were derived exclusively from the surface horizons (Ap1 and Ap2). A core objective of this methodology was to test whether easily accessible surface biological signatures can serve as reliable, practical diagnostic indicators for the whole‐profile soil health status, thereby enhancing the field applicability of the resulting models.

### 2.4 Microbiome Data Aggregation Strategies

To assess how different microbiome data representations affect the models' ability to predict soil characteristics, four distinct aggregation strategies were applied to the prokaryotic, fungal and microeukaryotic ASV tables. These strategies aimed to capture various compositional and ecological features of the soil microbiome.

In the first approach, the kingdom balance aggregation (ABS), the raw ASV data for the three microbial kingdoms (prokaryotes, fungi and microeukaryotes) were normalized to relative abundances and combined with absolute quantification data obtained by qPCR. This method provides a more ecological overview of the entire microbial diversity and more thoroughly captures the structural features of the microbial communities.

The second method, the centered log‐ratio (CLR) transformation, addressed the compositional nature of these data by applying this transformation (Aitchison 1982) to the abundance data of each kingdom. To handle zero values, a pseudo‐count of 1 was added prior to transformation (Equation 4). Prokaryotes, fungi and microeukaryotes were combined after the CLR transformation. This method aimed to capture the inherent compositional information within each microbial community, acknowledging that interactions between phyla and the presence of rare taxa can be critical. Consequently, the CLR transformation projects the data into Euclidean space, mitigating the spurious correlations inherent in relative abundance data and ensuring compatibility with standard machine learning algorithms:

(4) $$\mathrm{clr}\left(x_{i}\right)=\ln\left(\frac{x_{i}+1}{g\left(x+1\right)}\right)=\ln\left(x_{i}+1\right)-\frac{1}{D}\sum_{j=1}^{D}\ln\left(x_{j}+1\right)$$

where $x_{i}$ denotes the raw abundance (read count) of the i‐th ASV in the sample; the term +1 represents the pseudocount added to handle zero values prior to log‐transformation; D is the total number of ASVs (features) in the sample; and the term $\frac{1}{D}\sum_{j=1}^{D}\ln\left(x_{j}+1\right)$ corresponds to the arithmetic mean of the log‐transformed adjusted counts (equivalent to the natural logarithm of the geometric mean $g\left(x+1\right)$).

In the third method, taxonomic aggregation (TAXO), ASVs were aggregated to calculate the relative abundances of major taxonomic groups, primarily at the family level, for prokaryotes, fungi and microeukaryotes. This method highlights the contributions of broadly defined, abundant taxa, which are often linked to general ecological roles and soil processes.

In the fourth method, functional aggregation (FUNCTION), the functional potentials of bacterial ASVs were predicted from the 16S rRNA gene data using PICRUSt2 (Douglas et al. 2020). Generated KEGG Orthologs (KOs) were categorized into functional modules (Kanehisa and Goto 2000) to evaluate specific functions, such as nitrification or nitrogen fixation within nitrogen metabolism. For fungi, ecological guilds were assigned using FUNGuild (Nguyen et al. 2016). In this aggregation approach, microeukaryotic organisms were excluded because current predictive tools and databases lack the resolution required to accurately assess their functionality. This strategy directly links the microbial community to its potential functional capabilities relevant to the predicted soil properties, shifting the focus from taxonomic composition to potential ecological activity.

For each of these four aggregation methods, features were filtered to retain only those with a prevalence greater than 10% across all samples. The resulting microbial features were then combined with specific abiotic pedoclimatic variables to form the complete input feature set for the ML models, accounting for the physical and environmental context of the sampling sites. Following this integration, the final datasets contained 1513 predictors for the absolute abundances (ABS) strategy, 1471 for the centered log‐ratio (CLR) strategy, 514 for the taxonomic aggregation (TAXO) and 365 for the functional aggregation (FUNCTION). Specifically, the soil texture variables included as continuous predictors were the relative contents of clay, sand and silt. The climatic variables incorporated into the models were growing degree days (GDD), total precipitation and the Gini index of precipitation. Because the selected non‐parametric machine learning algorithms are inherently robust to multicollinearity, all three compositional texture fractions were included as predictors without prior exclusion.

### 2.5 Machine Learning Framework: Algorithms, Training, Validation and Model Selection

**FIGURE 2:**
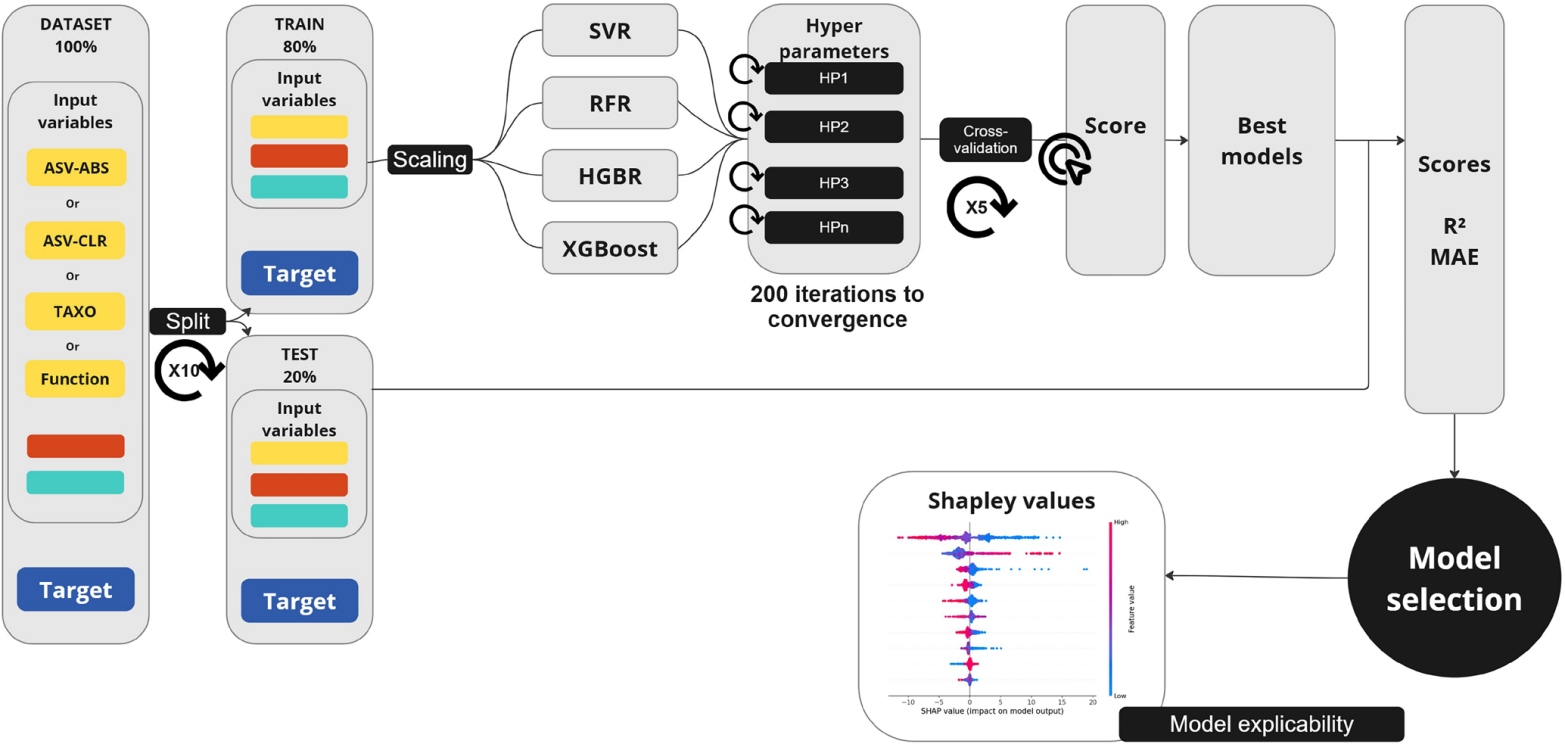
Workflow for the development and optimization of machine learning models to predict key soil functions. The process involved 10 iterations of a random 80/20 train/test split to assess model stability and generalization. Within each iteration, a Bayesian optimization approach was used for hyperparameter fine‐tuning of all four model types (HGBR, RFR, XGBoost and SVR), running for 200 iterations to achieve convergence. Model evaluation was performed by calculating the mean and standard deviation across iterations for two key performance metrics: The coefficient of determination ($R^{2}$) and the mean absolute error (MAE). Following the identification of the best‐performing model architecture, model explainability was assessed using Shapley Additive Explanations (SHAP). SHAP values were calculated to quantify the relative contribution and directional influence of individual microbial, textural and climatic features on the predicted ecological outcomes. This integrated pipeline ensured that ecological interpretations were derived only from the most robust and optimized predictive frameworks.

A set of commonly used supervised machine learning algorithms was tested for its ability to predict each of the three output soil properties (Figure 2). These included four established models: Random Forest Regression (RFR) (Breiman 2001), Support Vector Regression (SVR) (Drucker et al. 1996), Histogram Gradient Boosting Regression (HGBR) (Ke et al. 2017) and eXtreme Gradient Boosting Regression (XGBoost) (Chen 2016). The selection of these algorithms was based on their proven effectiveness in managing complex, non‐linear data and high‐dimensional feature spaces commonly encountered in soil science and ecological research (Wadoux et al. 2020; Topçuoğlu et al. 2020).

To maximize statistical power and preserve fine‐scale ecological relationships, the four spatial sampling points within each field, as well as the distinct Ap horizons, were maintained as independent observations rather than being averaged per site. Because soil texture, microbiome composition, bulk density and horizon thickness were measured individually at each point, this approach allowed the models to capture the significant intra‐field spatial heterogeneity driving point‐specific variations in SOC stock, MWD and macroporosity. Furthermore, given that tree‐based algorithms are inherently robust to multicollinearity, we retained the full suite of predictors (up to 1513 predictors after filtering) without performing prior correlation‐based feature reduction.

The dataset was randomly split into a training set (80% of samples) and an independent testing set (20% of samples). Hyperparameter tuning for each algorithm was performed using a 5‐fold cross‐validation scheme on the training set. We employed a Bayesian convergence search strategy (Snoek et al. 2012) to efficiently and systematically find the optimal hyperparameter combination that minimized the mean absolute error (MAE), thereby mitigating the risk of overfitting (Table S1).

Final model performance on the independent testing set was evaluated and reported using two key metrics: the coefficient of determination ($R^{2}$) and the mean absolute error (MAE). The $R^{2}$ value indicates the proportion of variance in the output variable explained by the model, providing a goodness‐of‐fit measure. The MAE represents the average absolute difference between predicted and actual values. While the root mean squared error (RMSE) is sometimes used in similar studies, we elected to report MAE as our primary metric of absolute error. MAE provides a more intuitive and unambiguous measure of average error magnitude in highly variable ecological datasets, avoiding the disproportionate inflation caused by single outliers typical of RMSE (Willmott and Matsuura 2005).

To evaluate the influence of the initial random data partition on result variability and model stability, 10 iterations of the entire training and testing process were conducted. For each output variable and each microbiome aggregation strategy, the machine learning algorithm that showed the best overall predictive performance on the test set (primarily based on the highest $R^{2}$ and lowest MAE) was chosen as the ‘best model’ for subsequent feature importance analysis using Shapley Additive Explanations (SHAP) values (Lundberg and Lee 2017).

### 2.6 Feature Importance: Application and Rationale for Shapley Additive Explanations (SHAP)

To interpret the best‐performing ML models and identify the most influential input features, an approach based on Shapley Additive Explanations (SHAP) was used. SHAP is a game theory‐based method that explains an instance's prediction by calculating each feature's contribution to it (Lundberg and Lee 2017). It offers both global feature importance (the overall impact of a feature across all samples) and local interpretability (the influence of a feature on a specific sample's prediction). For each prediction, SHAP values were calculated for each feature, representing its marginal contribution to the difference between the actual prediction and the average prediction. Positive SHAP values indicate that a feature contributes to increasing the predicted value, while negative values suggest a contribution to decreasing it. The magnitude of the SHAP value indicates the strength of this contribution. SHAP summary plots (beeswarm plots) were created to visualize global feature importance, showing the distribution of SHAP values for each feature. Concurrently, SHAP dependence plots illustrated how the model's output changes as a single input feature varies, highlighting potential interaction effects with other features (Figures S2–S4).

While Support Vector Regression (SVR) frequently achieved the highest primary predictive performance, it was excluded from the SHAP analysis due to its significant instability across different aggregation methods and the high computational intensity required for kernel‐based explainers. To evaluate feature importance efficiently and reliably, we utilized the tree‐based algorithms. Random Forest Regression (RFR) was excluded due to its comparatively lower predictive accuracy. Histogram Gradient Boosting Regression (HGBR) was ultimately selected over eXtreme Gradient Boosting Regression (XGBoost) because it provided the optimal balance: it achieved high $R^{2}$ scores while demonstrating superior stability (lower variance across iterations) and consistent performance across all omics aggregation strategies. Furthermore, to enable a standardized comparison of feature importance across all three distinct soil ecological functions, we applied the SHAP framework uniformly to the HGBR models utilizing the absolute abundances (ABS) aggregation strategy.

### 2.7 Statistical Analyses

Beyond standard machine learning evaluation metrics, statistical analyses were conducted to determine the significance of observed differences in model performance ($R^{2}$) using a two‐way analysis of variance (ANOVA). This model evaluated the main effects and the interaction between the machine learning algorithm and the microbiome data aggregation strategy. Furthermore, because all models were evaluated on the exact same 10 random train/test data splits, the iteration number was included in the ANOVA model as a blocking factor to account for the non‐independence of the repeated measures. Upon identifying significant interaction effects, a *post hoc* Duncan's multiple range test (α=0.05) was applied to the interaction terms to categorize the distinct predictive pipelines into statistically distinct groups. All machine learning workflows were executed using Python (version 3.11.2) with libraries including scikit‐learn, SHAP, pandas and NumPy. Statistical testing and visualization were performed in R (version 4.3.1), utilizing the ggplot2 package for graphics and the agricolae package for Duncan's test.

## 3 Results

We built machine learning models to predict three key ecological soil functions, namely soil organic carbon (SOC) stock, macroporosity (MPR) and soil aggregate size and stability (evaluated as MWD). Using both abiotic variables (e.g., soil texture and climate) and diverse soil microbiome data as predictors, we investigated the impact of different microbiome data aggregation methods on model performance.

### 3.1 ASV‐Based Aggregations Outperformed Taxonomic and Functional Approaches as Predictors

Machine learning models showed an interesting overall performance for the three target soil properties, with $R^{2}$ varying between 0.562 and 0.819 depending on the algorithm and the type of omics data aggregation. The performance metrics for the best‐performing models (developed via hyperparameter tuning, cross‐validation and evaluation on the independent test set) for each output variable are summarized in Table 1.

**TABLE 1:** Predictive performance metrics for the three target soil functions. Values represent the mean and standard deviation evaluated on the independent testing sets across 10 random train/test split iterations for soil organic carbon (SOC) stock, mean weight diameter (MWD) and macroporosity (MPR). Machine learning models evaluated include Histogram Gradient Boosting Regression (HGBR), Random Forest Regression (RFR), Support Vector Regression (SVR) and eXtreme Gradient Boosting Regression (XGBoost). MAE: Mean absolute error (Units—SOC stock: Mg ha^−1^; MWD: Mm; MPR: Cm^3^ cm^−3^); $R^{2}$: coefficient of determination. Omics data aggregation strategies: Absolute abundances (ABS), centered log‐ratio (CLR) transformation, functional aggregation (FUNCTION) and taxonomic aggregation (TAXO).

Target	Model	Aggregation	MAE	MAE (SD)	$R^{2}$	$R^{2}$ (SD)
SOC	HGBR	ABS	16.423	0.460	0.673	0.025
SOC	HGBR	CLR	16.183	0.489	0.680	0.015
SOC	HGBR	FUNCTION	17.953	0.633	0.611	0.027
SOC	HGBR	TAXO	17.526	0.801	0.629	0.029
SOC	RFR	ABS	18.219	0.815	0.606	0.036
SOC	RFR	CLR	18.250	0.993	0.597	0.030
SOC	RFR	FUNCTION	18.400	0.607	0.596	0.016
SOC	RFR	TAXO	18.593	0.834	0.572	0.021
SOC	SVR	ABS	16.365	0.442	0.632	0.038
SOC	SVR	CLR	15.772	0.506	0.706	0.022
SOC	SVR	FUNCTION	19.513	0.812	0.543	0.038
SOC	SVR	TAXO	19.178	0.625	0.562	0.056
SOC	XGBoost	ABS	16.903	0.827	0.657	0.027
SOC	XGBoost	CLR	16.335	0.725	0.678	0.020
SOC	XGBoost	FUNCTION	18.318	0.580	0.611	0.025
SOC	XGBoost	TAXO	17.656	0.636	0.624	0.014
MWD	HGBR	ABS	0.427	0.014	0.717	0.014
MWD	HGBR	CLR	0.425	0.012	0.703	0.021
MWD	HGBR	FUNCTION	0.464	0.019	0.666	0.031
MWD	HGBR	TAXO	0.456	0.010	0.677	0.026
MWD	RFR	ABS	0.462	0.015	0.669	0.029
MWD	RFR	CLR	0.472	0.015	0.671	0.021
MWD	RFR	FUNCTION	0.477	0.015	0.649	0.024
MWD	RFR	TAXO	0.480	0.012	0.643	0.019
MWD	SVR	ABS	0.435	0.017	0.667	0.100
MWD	SVR	CLR	0.408	0.013	0.735	0.010
MWD	SVR	FUNCTION	0.472	0.017	0.635	0.037
MWD	SVR	TAXO	0.476	0.020	0.571	0.149
MWD	XGBoost	ABS	0.456	0.025	0.668	0.021
MWD	XGBoost	CLR	0.461	0.023	0.667	0.028
MWD	XGBoost	FUNCTION	0.482	0.017	0.648	0.034
MWD	XGBoost	TAXO	0.479	0.022	0.643	0.035
MPR	HGBR	ABS	0.022	0.000	0.788	0.016
MPR	HGBR	CLR	0.022	0.001	0.781	0.015
MPR	HGBR	FUNCTION	0.023	0.001	0.756	0.022
MPR	HGBR	TAXO	0.023	0.001	0.758	0.022
MPR	RFR	ABS	0.024	0.001	0.741	0.015
MPR	RFR	CLR	0.024	0.001	0.746	0.020
MPR	RFR	FUNCTION	0.024	0.001	0.749	0.015
MPR	RFR	TAXO	0.024	0.001	0.739	0.016
MPR	SVR	ABS	0.021	0.001	0.707	0.189
MPR	SVR	CLR	0.021	0.001	0.819	0.011
MPR	SVR	FUNCTION	0.024	0.001	0.740	0.015
MPR	SVR	TAXO	0.024	0.001	0.706	0.054
MPR	XGBoost	ABS	0.023	0.001	0.735	0.014
MPR	XGBoost	CLR	0.024	0.001	0.733	0.023
MPR	XGBoost	FUNCTION	0.025	0.001	0.721	0.024
MPR	XGBoost	TAXO	0.024	0.001	0.722	0.029

**FIGURE 3:**
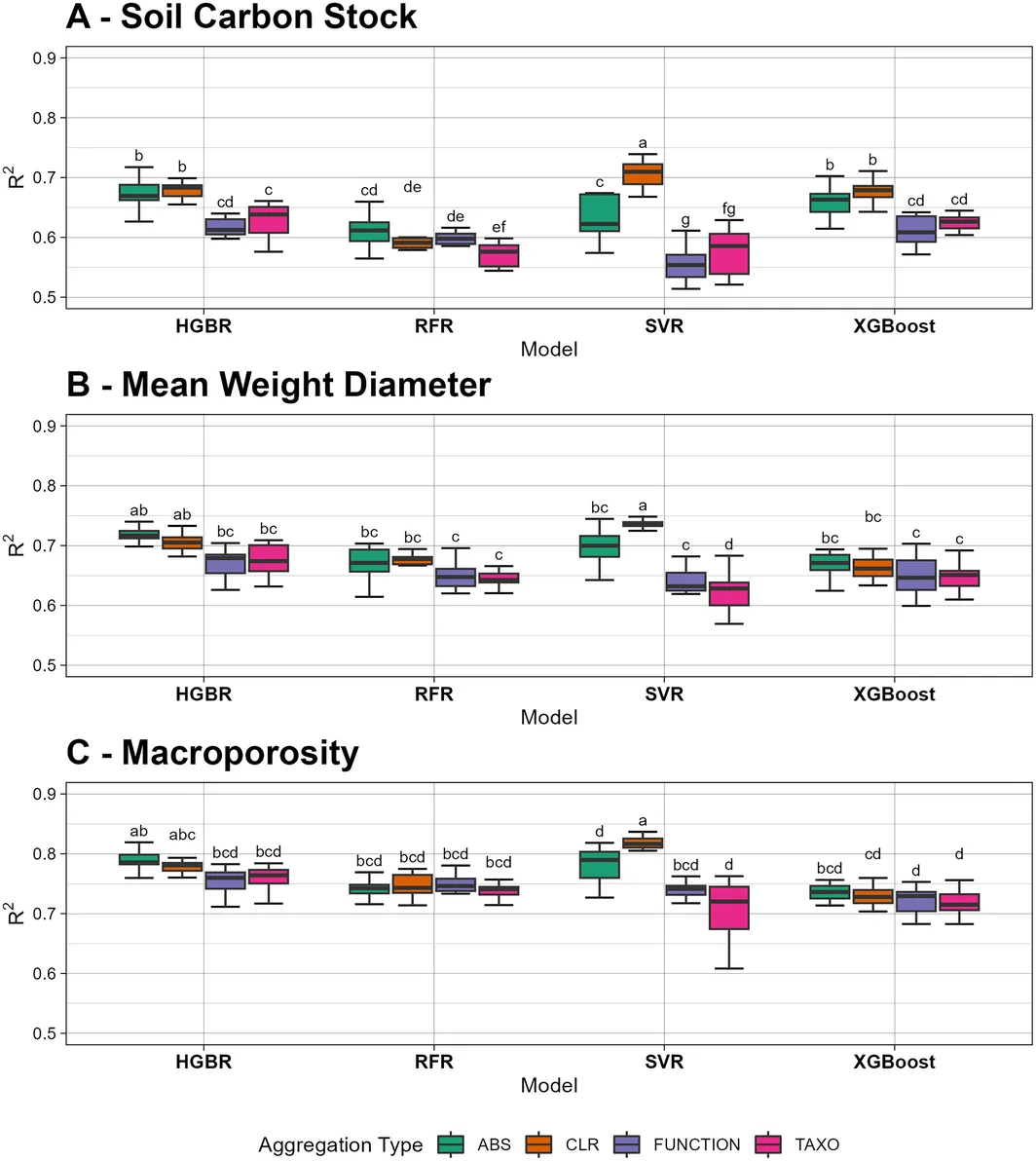
Comparison of model performances for predicting three key soil functions based on data aggregation type. The performances of four distinct machine learning algorithms, Histogram Gradient Boosting Regression (HGBR), Random Forest Regression (RFR), Support Vector Regression (SVR) and eXtreme Gradient Boosting Regression (XGBoost), were compared for the prediction of three separate soil functions: Soil organic carbon (SOC) stock (Panel A), mean weight diameter (MWD) (Panel B) and macroporosity (Panel C). Model performance is expressed as the coefficient of determination, $R^{2}$, between the predicted and observed values (y‐axis). Boxplots display the distribution of $R^{2}$ values for each algorithm, evaluated on the independent testing sets across 10 random train/test split iterations, under four input omics data aggregation strategies: Absolute abundances (green), centered log‐ratio transformation (orange), functional aggregation (blue) and taxonomic aggregation (pink). Statistical significance of the interaction between the machine learning model and the aggregation strategy was assessed using Duncan's multiple range test following a two‐way ANOVA, with iteration included as a blocking factor (α=0.05). A corresponding evaluation of the mean absolute error (MAE), quantifying the specific prediction error rates for all models and strategies, is provided in Figure S1.

The different models and algorithms globally enabled the prediction of soil organic carbon (SOC) stock with high $R^{2}$ values, suggesting robust predictive performance for this function. The SVR model combined with the CLR data aggregation (Figure 3A) achieved the highest performance, which was statistically superior to all other combinations (group ‘a’), yielding a median $R^{2}$ value approaching 0.70. The HGBR and XGBoost models showed robust and acceptable performances, with $R^{2}$ values predominantly ranging between 0.65 and 0.68. The RFR model was slightly weaker than the other three. The impact of the aggregation choice was particularly pronounced for the SVR model, where FUNCTION and TAXO aggregations (groups ‘fg’ and ‘g’) led to a significant performance drop compared to ABS and CLR aggregations, while exhibiting greater variability than the other algorithms.

The mean weight diameter (MWD), which indicates the average level of soil aggregation, was predicted with higher accuracy compared to the SOC stock, yielding $R^{2}$ values ranging from 0.57 to 0.73 (Figure 3B). The SVR model with ABS aggregation (group ‘a’) again emerged as the top performer (median $R^{2}$ of 0.735). However, the performance gap between this SVR model and the top HGBR models (groups ‘ab’) was less pronounced. The HGBR models demonstrated highly stable performance across the four aggregation types. Similar to the SOC stock predictions, the SVR model was the most sensitive to the aggregation method, exhibiting significantly lower performance for the FUNCTION type and, notably, the TAXO type (group ‘d’).

Finally, model performance was highest for macroporosity predictions, achieving $R^{2}$ values notably higher than for the other two soil characteristics, mainly ranging from 0.70 to 0.82 (Figure 3C). The best approach was again the SVR with CLR aggregation (orange), which achieved the highest score with a median $R^{2}$ of 0.819. The HGBR model showed comparable performance between the CLR and ABS aggregations, with no statistically significant differences. For nearly all models, the FUNCTION and TAXO aggregations tended to yield the lowest results ($0.706<R^{2}<0.758$), highlighting the difficulty of accurately predicting this physical soil function using coarse taxonomic or functional groupings.

Altogether, the model comparison analysis revealed a clear duality. On one hand, the SVR models achieved the highest peak predictive performances across all three soil ecological functions, especially when combined with the CLR aggregation strategy. However, SVR performance was relatively unstable, varying significantly depending on the aggregation method and showing greater fluctuation across cross‐validation runs (as indicated by the boxplots in Figure 3 and the corresponding standard deviations). On the other hand, the Histogram Gradient Boosting Regression (HGBR) model, paired with ABS or CLR aggregations, provided an optimal balance between high predictive capability and robustness. Its $R^{2}$ scores were consistently high and frequently approached the peak SVR scores, yet it offered notably superior stability across various aggregation strategies and lower variability across the different train/test split iterations.

### 3.2 Identifying Important Features Using Shapley Additive Explanations (SHAP)

Based on its optimal balance of high predictive performance and stability across all three ecological functions (as established in Section 3.1), the HGBR model coupled with the ABS aggregation strategy was selected to generate SHAP values. The application of this SHAP framework allowed for the identification and quantification of the most influential abiotic and microbial features driving the predictions for each target variable.

#### 3.2.1 Fungal Features Contributed Negatively to SOC Stock Predictions

**FIGURE 4:**
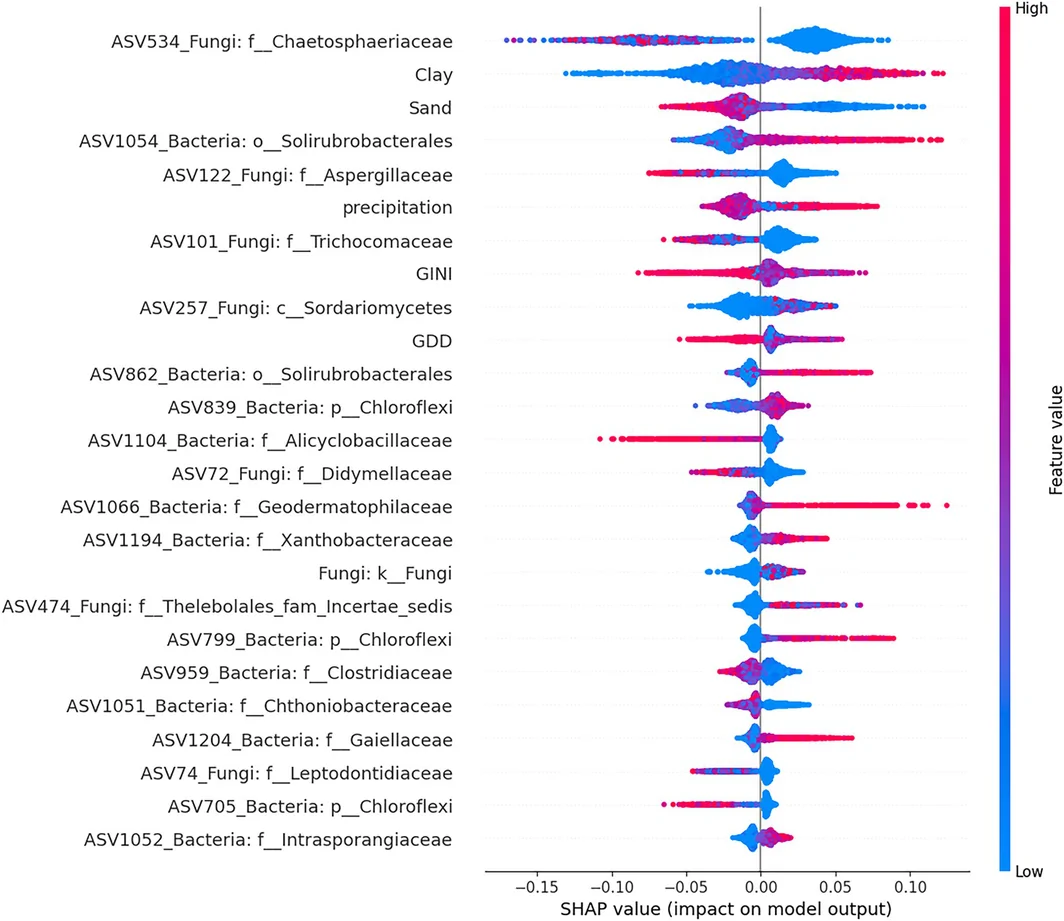
SHAP beeswarm plot for the soil organic carbon (SOC) stock model. The plot displays the influence of the top‐ranking features on the model output. Each row represents a feature, ranked by its mean absolute SHAP value (global importance). Each point represents an individual soil sample, with the colour indicating the feature's relative value (red = high, blue = low). The horizontal position (x‐axis) indicates the SHAP value, representing the magnitude and direction of the feature's impact on the SOC stock prediction. Positive SHAP values indicate an increase in predicted SOC stock.

As shown in the beeswarm plot for SOC stock (Figure 4), the most influential features included both fungal and bacterial attributes, along with key soil texture and climatic variables. As expected, clay content correlated positively with the predicted SOC stock. Climatic variables, such as GDD and the Gini index of precipitation, also contributed to the model, mostly exhibiting negative contributions. Their distribution was less spread out compared to clay content and displayed a less linear relationship, likely due to numerous interaction effects. Interestingly, microbial features were also among the top contributors to the model, with three of the four most contributing ASVs being fungi: Chaetosphaeriaceae (ASV534), Aspergillaceae (ASV122) and Trichocomaceae (ASV101). Their high occurrence was linked to a substantial decrease in SOC stock content, suggesting a strong negative correlation with the predicted SOC stock. However, other influential fungal features, such as Sordariomycetes (ASV257) and Thelebolales (ASV474), were positively associated with SOC stock.

Conversely, almost all important bacterial features (9 out of 12 bacterial ASVs among the top 25 features) had a general positive influence on the model's results. The most important bacterial feature, Solirubrobacterales (ASV1054), displayed a clear, well‐distributed pattern positively associated with SOC stock.

#### 3.2.2 Clay Content and Bacterial Signatures Dominate Positive MWD Predictions

**FIGURE 5:**
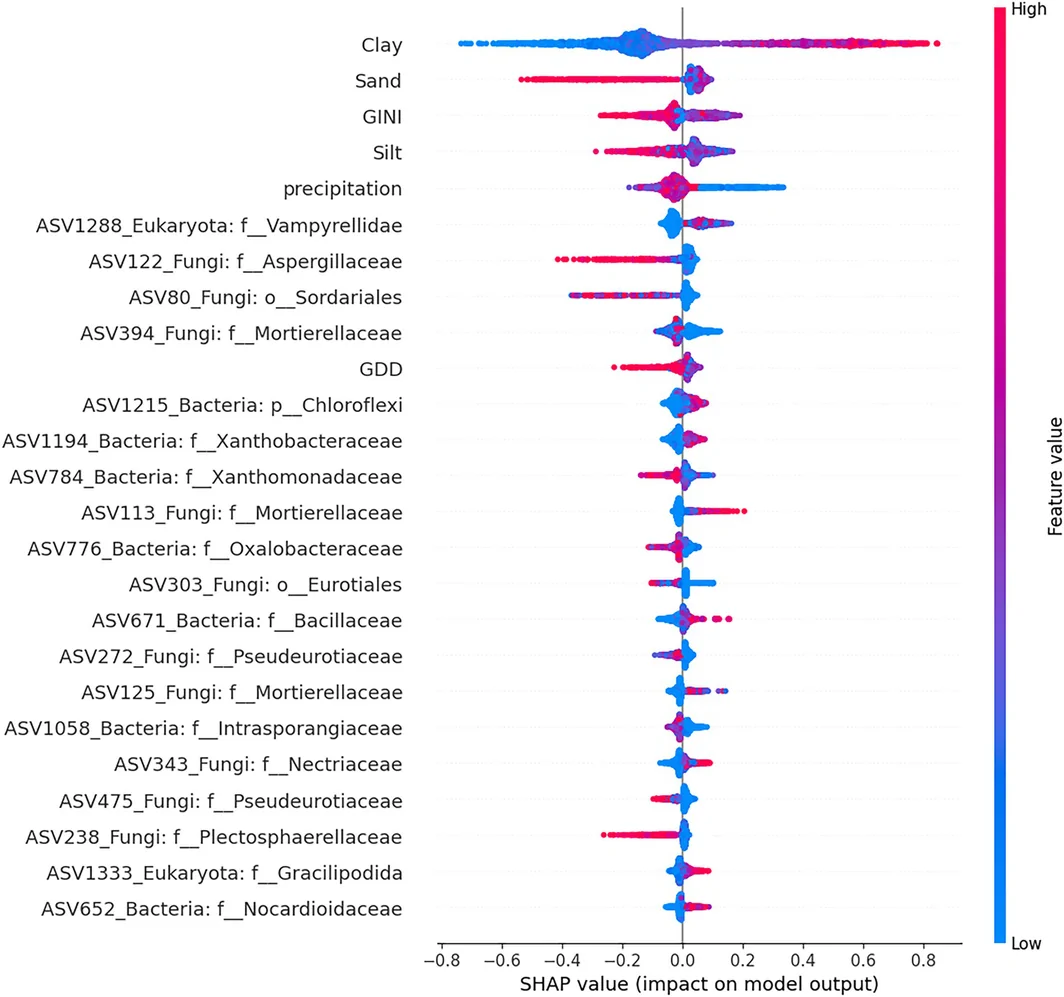
SHAP beeswarm plot for the mean weight diameter (MWD) model. The plot displays the top‐ranking features influencing the model's prediction of MWD, ranked by mean absolute SHAP value. For details on plot interpretation and colour coding, refer to the caption of Figure 4.

Regarding the MWD model, the SHAP values analysis highlighted the dominant contribution of physical and climatic variables (Figure 5). Similar to the SOC stock, clay content was strongly linked to increased predicted MWD. In contrast, the Gini index, total precipitation and GDD showed negative associations with the predicted MWD. Microbial features then contributed positively, including a microeukaryotic Vampyrellidae feature (ASV1288) and several bacterial features: Chloroflexi (ASV1215), Xanthobacteraceae (ASV1194), Bacilliaceae (ASV671) and Nocardioidaceae (ASV652). Conversely, fungal features were mainly negatively correlated with the predicted MWD: Aspergillaceae (ASV122), Sordariales (ASV80), Pseudeurotiaceae (ASV272, ASV475) and Plectosphaerellaceae (ASV238).

#### 3.2.3 Interplay of Abiotic and Microbial Signatures in Macroporosity Predictions

**FIGURE 6:**
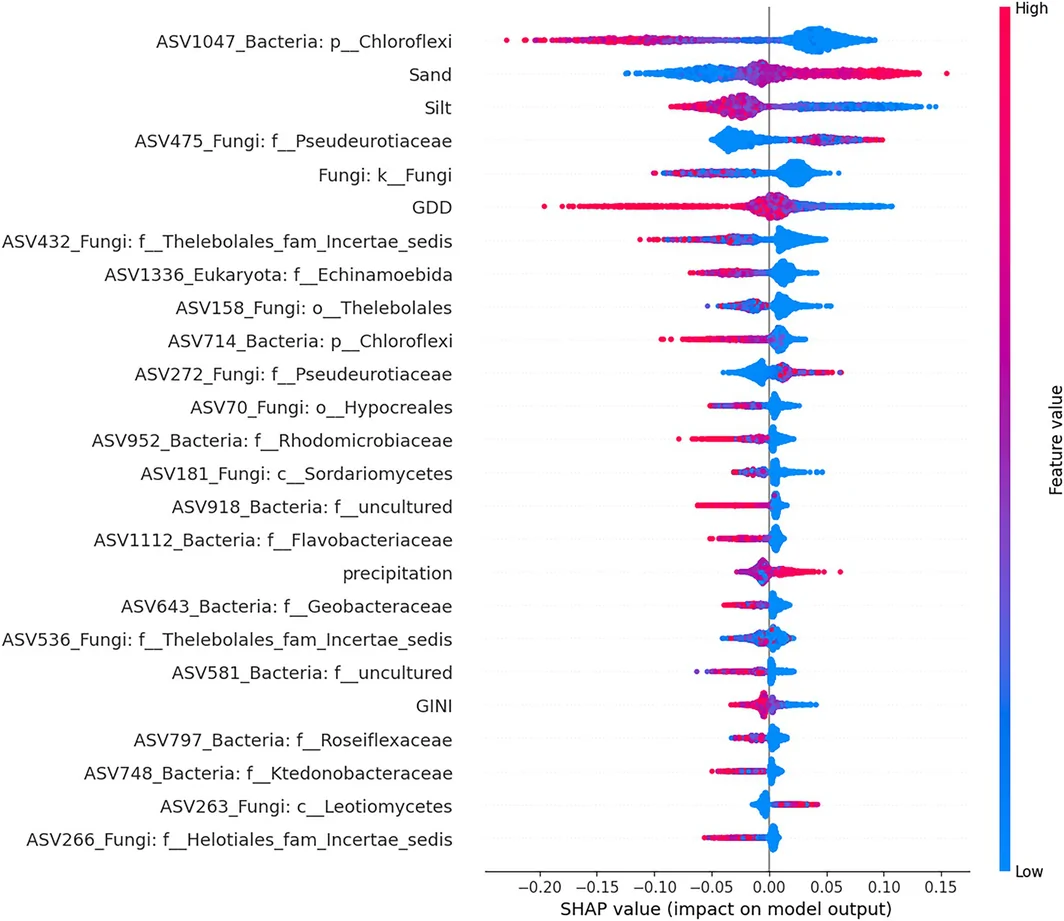
SHAP beeswarm plot for the macroporosity model. The plot displays the top‐ranking features influencing the model's prediction of macroporosity, ranked by mean absolute SHAP value. For details on plot interpretation and colour coding, refer to the caption of Figure 4.

SHAP analysis of the macroporosity model revealed a strong interplay between abiotic architectural constraints and specific microbial features (Figure 6). Because macroporosity was weighted by horizon thickness and bulk density to reflect profile‐scale architecture, textural features emerged as primary drivers. Specifically, sand content was among the top overall contributors and exhibited a strong positive association, while silt and climatic variables such as growing degree days (GDD) were also highly influential but negatively associated.

Biologically, the model was dominated by specific prokaryotic and fungal predictors, primarily showing negative associations with macroporosity. The single most impactful predictor was a bacterial feature, Chloroflexi (ASV1047), which strongly decreased the predicted macroporosity. Other notable bacterial features, including Chloroflexi (ASV714) and Geobacteraceae (ASV643), also exhibited negative SHAP values. Fungal predictors displayed contrasting effects: specific taxa like Pseudeurotiaceae (ASV475) were positively associated, whereas Thelebolales (ASV432) exhibited a negative impact. The microeukaryotic signal was distinctly represented by Echinamoebida (ASV1336), which showed a strong negative association. The distribution patterns of these top biological features were characterized by a broad dynamic range, indicating high sensitivity to variations in the physical pore network.

## 4 Discussion

### 4.1 The Predictive Power of the Microbiome in Soil Systems

The strong predictive performance of our best‐optimized models for soil organic carbon (SOC) stock, mean weight diameter (MWD) and macroporosity (with peak $R^{2}$ values ranging from 0.70 to 0.82 across the three functions) highlights the critical role of the microbiome in soil physical functioning. Traditionally, estimating these hydraulic and structural properties has relied on pedotransfer functions. These are typically linear parametric equations that infer structure primarily from static abiotic variables like soil texture and bulk density (Weber et al. 2024). However, the prominent contribution of microbial features to our predictive models, as evidenced by the SHAP analysis, reinforces the concept that soil structure is not merely a static geological feature, but a dynamic property driven by biological agents. While texture provides the geological framework that defines the physical limits of a soil system, it is biological activity that organizes this framework into a functional architecture (Young and Ritz 2000). Consequently, static abiotic variables alone cannot reflect the dynamic effects of seasonal shifts or agricultural management. While microbial data does not replace explicit management records, the microbiome acts as an integrative biological sensor that responds rapidly to these fluctuations. By incorporating microbial signatures, we capture the proximate biophysical drivers of soil function, such as bacterial exopolysaccharide production and microbial necromass accumulation (mechanisms detailed specifically for our top predictors in Section 4.3), that static abiotic baselines inherently miss. The microbiome thus serves as a dynamic biological indicator of structural stability, providing the granularity needed to explain the heterogeneity of soil architecture (Vereecken et al. 2016).

Furthermore, the strong predictive performance of our non‐parametric models, specifically Histogram Gradient Boosting Regression (HGBR) and Support Vector Regression (SVR), is consistent with their ability to capture highly non‐linear coupling between microbial communities and soil physical properties. For instance, the stabilizing effect of extracellular polymeric substances (EPS) often exhibits saturation thresholds or relies on strong organo‐mineral interactions with clay surfaces (Totsche et al. 2018), complex dependencies that simple linear coefficients often fail to capture. Additionally, high‐dimensional microbiome datasets are characterized by sparsity and multi‐way feature interactions that violate the assumptions of traditional linear pedometrics (e.g., multiple linear regression). The success of these advanced algorithms in accommodating this complexity aligns with the methodological findings of Hermans et al. (2020), who demonstrated that nonlinear frameworks are essential for extracting functional signals from soil DNA. Ultimately, our results reinforce the shift proposed by Wadoux et al. (2020) to move beyond traditional linear pedometrics toward models capable of resolving complex, interactive biotic signals.

### 4.2 Limitations of Data Aggregation

Aggregating ASVs to the family level consistently decreased predictive power, challenging the assumption of functional redundancy in soil physical modification. This reduction in signal indicates that traits governing soil architecture, such as the secretion of specific binding polysaccharides or hyphal strength, are not phylogenetically conserved across broad taxonomic groups. Instead, these complex environmental adaptations appear restricted to specific lineages at the species or strain level, aligning with the framework proposed by Martiny et al. (2015). Consequently, broad taxonomic aggregation obscures the role of rarer biodiversity, where low‐abundance but high‐impact architects may be averaged into larger, dominant groups.

This signal dilution is compounded by the inherent limitations of current taxonomic reference databases (such as SILVA and UNITE). Since a large portion of soil microbial diversity remains uncultured or taxonomically unassigned (Delgado‐Baquerizo et al. 2018), coarse‐graining forces distinct, uncharacterized organisms into artificial clusters. This hides the fine‐scale ecological differences inherently captured by amplicon sequence variants (ASVs), which are resolved independently of taxonomic databases (Callahan et al. 2017). Furthermore, this taxonomic compression limits the efficacy of functional inference tools like PICRUSt2 and FUNGuild. Because these tools rely on the same incomplete reference frameworks to predict gene content, they struggle to resolve the highly specific biophysical traits that directly construct soil architecture, such as the adhesive viscosity of exopolysaccharides that cement micro‐aggregates, or the morphology of filamentous bacteria that physically enmesh them (Tisdall and Oades 1982; Costa et al. 2018).

By maintaining ASV‐level resolution, our models successfully bypassed these limitations to isolate specific biological engineers. As evidenced by our SHAP analyses (e.g., Figures 4 and 5), the highest predictive importance was attributed to discrete entities such as ASV1194 (Xanthobacteraceae, known EPS producers) and ASV1215 (filamentous Chloroflexi). Because critical architectural traits are highly specific, grouping these actual soil engineers with non‐engineering relatives at higher taxonomic ranks dilutes their biophysical signal. This mechanism directly explains the reduction in predictive performance ($R^{2}$) observed in our taxonomically aggregated models. Supporting the methodological recommendations outlined by Knight et al. (2018), our results confirm that high‐resolution ASV data is critical for capturing the specific biological variations that drive the fine‐scale heterogeneity of soil microhabitats.

The systematically lower predictive power obtained when using taxonomic aggregation in machine learning approaches reinforces that the functional traits governing soil structure are not widely distributed, but rather specific to distinct microbial guilds. This highlights the necessity of high‐resolution approaches to examine how specific taxa drive the hierarchical development of soil—from chemical stabilization to physical architecture, as discussed below.

### 4.3 Microbial Signatures Act as Complex Engineers

Our results indicate a functional hierarchy in the organization of the soil matrix. Specific prokaryotic signatures strongly predict the total soil organic carbon (SOC) stock, an accumulation likely driven by their documented roles in biochemical stabilization (e.g., via necromass). While this biochemical foundation acts as a precursor to the physical stabilization of aggregates (MWD), the prediction of macroporosity relies on distinct microeukaryotic signals. This supports a model of biological hierarchy where microbial activity first establishes a chemical foundation, then engineers physical stability through bacterial scaffolding and trophic regulation and finally responds to the physical constraints imposed by the pore network, such as water retention and the continuity of aqueous films.

#### 4.3.1 Microbial Drivers of Carbon Stabilization

The predictive relationship between bacterial signatures and SOC stock suggests a prokaryotic‐driven accumulation pathway. This aligns with the microbial efficiency‐matrix stabilization framework (Cotrufo et al. 2013), which posits that stable soil carbon originates from efficient microbial processing rather than the selective preservation of recalcitrant plant compounds. The SHAP analysis identified Solirubrobacterales (ASV1054) as a primary positive predictor of SOC stock. As Actinobacteriota are characterized by high carbon use efficiency and metabolic versatility (Herman et al. 2012), their dominance implicates the microbial carbon pump (Liang et al. 2017) as a driver of accumulation. These bacteria assimilate labile carbon, generating a precursor pool of necromass that binds to mineral surfaces to form stable mineral‐associated organic matter (Kallenbach et al. 2016; Cotrufo et al. 2013). Conversely, the negative contribution of fungal taxa such as Aspergillaceae (ASV122) and Chaetosphaeriaceae (ASV534) indicates a destabilizing role. These fungi act as efficient decomposers, likely mining stable organic matter for nutrients (Dignam et al. 2019). Their negative association with SOC stock suggests that their activity favours respiratory loss over stabilization, reflecting a system‐level trade‐off between bacterial anabolism (necromass formation) and fungal catabolism (mineralization).

#### 4.3.2 Biological Mechanisms of Aggregate Formation

The prediction of mean weight diameter suggests an alternative aggregation mechanism to the classic aggregate hierarchy model (Tisdall and Oades 1982), which posits fungal hyphae as the primary temporary binding agents. Our models showed that specific fungal signatures were negatively correlated with aggregation, whereas bacterial and microeukaryotic features were positive predictors. While it is plausible that fungal abundance generally correlates with sandy, poorly aggregated soils due to environmental filtering, our machine learning models simultaneously incorporate abiotic variables (e.g., sand and clay content) as predictive features. Therefore, the negative SHAP values for these specific fungal taxa represent an active destabilizing biological effect, rather than merely a passive covariation with sandy soil texture. As suggested by their taxonomy, these taxa are primarily saprotrophic decomposers rather than arbuscular mycorrhizal fungi (AMF); their activity likely favours the enzymatic breakdown of organic binding agents (EPS) rather than physical enmeshment. This implies that aggregation in these specific soils is supported instead by prokaryotic scaffolding and trophic protection. Positive bacterial predictors, including Chloroflexi (ASV1215) and Xanthobacteraceae (ASV1194), likely function as binding agents. Filamentous Chloroflexi can form dense, extensive structures in biofilms (Speirs et al. 2019), potentially substituting for fungal hyphae in holding micro‐aggregates together. Similarly, Xanthobacteraceae are known producers of exopolysaccharides (EPS) (Costa et al. 2018; Oren 2014), which act as mucilaginous cements for soil particles. The positive contribution of the mycophagous protist Vampyrellidae (ASV1288) provides evidence for top‐down regulation. Vampyrellidae perforate and consume fungal cells (Hess et al. 2012). Given the negative association of fungi with MWD in our results, Vampyrellidae may stabilize aggregates by exerting predatory pressure on fungal decomposers that would otherwise disrupt soil structure. This points to a trophic cascade in which protists protect soil physical structure by regulating the population of fungal destabilizers.

#### 4.3.3 Environmental Filtering of the Pore Network

The prediction of profile‐scale macroporosity, weighted by horizon thickness and bulk density, reveals a clear hierarchical relationship: foundational soil texture dictates the physical architecture, which in turn acts as a strict environmental filter for the microbiome. As expected from soil physics, sand and silt contents emerged as the dominant abiotic predictors, establishing the baseline volume of large, rapidly draining pores. However, the strong predictive power of specific microbial taxa over this abiotic baseline highlights their role as highly sensitive biosensors of the soil's hydrological and aeration status.

The negative association of specific bacterial taxa with macroporosity provides evidence of micro‐environmental filtering driven by aeration and resource diffusion. The selection of Geobacteraceae (ASV643) as a negative predictor biologically validates the physical data. Members of the Geobacteraceae family are strict anaerobes that thrive in waterlogged, hypoxic environments (Lovley et al. 2011). Soils with high macroporosity facilitate rapid drainage and deep oxygen diffusion, creating a hostile, aerated environment that competitively excludes these anaerobic taxa. Similarly, the strong negative impact of Chloroflexi lineages (ASV1047, ASV714) likely reflects their ecological niche. Many soil Chloroflexi are oligotrophic, filamentous bacteria adapted to low‐resource environments; their filamentous morphology allows them to bridge and exploit tight micropores where diffusion is restricted (Speirs et al. 2019), making their abundance a reliable proxy for highly compacted or micropore‐dominated structures.

The microeukaryotic signal, specifically the negative association of an amoeba belonging to the Echinamoebida order (ASV1336), confirms that pore dimensions act as a physical filter for soil fauna (Geisen et al. 2018). Naked amoebae require a continuous network of aqueous films for locomotion, prey capture and survival (Clarholm 1981). In highly macroporous soils, rapid water drainage disrupts this aqueous continuum, isolating aquatic predators and increasing desiccation stress. Conversely, microporous matrices retain sufficient moisture tension to sustain these habitable water films. Ultimately, the integrated SHAP profile for macroporosity illustrates a clear ecological dynamic: the abiotic matrix sets the spatial constraints, while the surviving microbiome reflects the resulting hydrological and oxygen regimes.

### 4.4 Limitations and Future Directions

Despite the robust predictive performance of our models ($R^{2}$ up to 0.82), the machine learning framework employed here remains correlative. The identification of predictor taxa does not establish mechanistic causality, particularly given the distinction between potential ecosystem engineers (e.g., bacteria driving aggregation) and bio‐indicators (e.g., microeukaryotes selected by pore size). Differentiating these causal roles requires validating these *in silico* associations through controlled microcosm experiments with sterile soil inoculation to isolate specific biophysical contributions. Methodologically, DNA‐based sequencing captures dormant cells and relic DNA from dead organisms, making it difficult to isolate only the active microbial community. However, in the context of soil architecture, this persistence is an analytical advantage rather than a source of noise. Because soil organic carbon (SOC) stock and aggregate stability are cumulative properties driven largely by microbial necromass accumulation (Cotrufo et al. 2013), the total DNA pool provides a more accurate proxy for these historical legacy effects than transcriptomics, which captures only active metabolic states. While our machine learning framework successfully decoded these interactions within the studied temperate agricultural systems, future research must evaluate these models across contrasting pedoclimatic regions to define their domain of applicability. Specifically, applying this approach to highly weathered tropical soils (e.g., Oxisols, dominated by iron oxides and 1:1 clays) or volcanic soils (e.g., Andisols, characterized by amorphous allophane) will determine whether the specific microbial engineering traits identified here are universally conserved, or if the predictive biomarkers shift in response to fundamentally different mineralogical templates.

From a practical standpoint, classical pedotransfer functions are typically developed to predict expensive or difficult‐to‐measure soil properties using readily available and inexpensive data, such as soil texture or bulk density. In contrast, incorporating high‐throughput microbiome sequencing as predictive features currently demands greater financial and technical resources than directly measuring the target biophysical variables. Therefore, the primary utility of our machine learning framework is not to serve as a routine predictive pedotransfer function, but rather as an exploratory, interpretable tool for ecological characterization. By treating the multi‐kingdom microbiome as a high‐resolution bio‐indicator, these models uncover the complex biological drivers of soil architecture. Nevertheless, as sequencing technologies continue to democratize and decrease in cost, molecular profiling is progressively shifting from a fundamental research tool to a feasible diagnostic indicator for future agricultural soil health monitoring programs.

Furthermore, expanding this framework to encompass different land‐use strategies and management practices will provide critical insights into soil structural resilience. Because our models were trained exclusively on agricultural soils subjected to regular mechanical disturbance, the selected biological predictors likely reflect a high‐disturbance equilibrium. Frequent tillage physically severs fungal hyphal networks, which favours bacterial traits, such as EPS scaffolding by Xanthobacteraceae or biofilms by Chloroflexi, as the primary drivers of aggregate stability. In undisturbed ecosystems (such as forests or permanent pastures) or under no‐till management, we hypothesize a fundamental shift in predictive biomarkers. Without mechanical disruption, fungal networks could permanently establish and re‐emerge as dominant positive predictors of macroaggregation. Similarly, the absence of tillage compaction would allow for the establishment of stable biopores, shifting the drivers of macroporosity away from strict abiotic textural constraints toward biological engineering signals.

Finally, the specific biological hierarchy identified by our models raises important questions regarding the vulnerability of soil architecture to extreme climate events. We hypothesize that intense precipitation, severe droughts and freeze–thaw cycles would likely decouple the layers of this hierarchy. Rapid desiccation or mechanical disruption from freezing could immediately degrade bacterial biofilms, sever EPS linkages and disrupt the sensitive micro‐food web (e.g., Vampyrellidae), thereby crashing the structural stability (MWD). Concurrently, extreme flooding or drying of the pore networks would alter the physical habitat constraints for aquatic microfauna (e.g., Echinamoebida), destabilizing the biological indicators of macroporosity. However, the biochemical foundation, the SOC stock, stabilized by prokaryotic and fungal pathways alongside organo‐mineral interactions, would likely exhibit greater resilience and thermal inertia in the short term, as it is chemically protected rather than merely physically structured. Testing the differential resilience of these predictive biomarkers under climatic extremes represents a crucial next step in applying multi‐kingdom machine learning to soil conservation.

## 5 Conclusion

The physical organization of soil acts as a biologically mediated hierarchy rather than a passive byproduct of abiotic weathering. By aggregating ASV‐level signatures balanced by kingdom quantification, we recovered functional signals typically masked by the numerical dominance of bacteria. This weighted integration reveals a clear functional partitioning in how microorganisms interact with the soil matrix. While abiotic constraints such as soil texture establish the baseline physical limits for both aggregation and porosity, microbial lineages play distinctly different roles across these scales. For organic carbon accumulation and physical stabilization (SOC stock and MWD), prokaryotes, fungi and specific microeukaryotes act as active engineers through processes such as necromass accumulation, biopolymer secretion and trophic regulation. In contrast, the macro‐architecture of the soil (macroporosity) functions primarily as a strict environmental filter, where specific taxa, such as anaerobic bacteria and naked amoebae, serve as highly sensitive bio‐indicators of the resulting aeration and hydrological connectivity. These mechanisms validate soil structure as an ‘extended phenotype’ of the microbiome. Since static pedotransfer functions cannot capture these biological feedbacks, we propose a shift toward a dynamic ‘biotransfer’ framework. Multi‐kingdom omic aggregation thus offers a robust basis for predicting soil physical resilience against the pressures of climate instability and land‐use intensification.

## Author Contributions

**Thomas Jeanne:** conceptualization, methodology, software, formal analysis, data curation, visualization and writing – original draft. **Julien Prunier:** conceptualization, methodology, software and writing – review and editing. **Richard Hogue:** resources, project administration, writing – review and editing and funding. **Arnaud Droit:** conceptualization, resources, writing – review and editing, supervision and funding.

## Funding

This project was made possible in part thanks to funding from the Quebec Ministry of Agriculture, Fisheries and Food (Ministère de l'Agriculture, des Pêcheries et de l'Alimentation du Québec; MAPAQ). We acknowledge the support of the Natural Sciences and Engineering Research Council of Canada (NSERC). The funders had no role in study analysis, decision to publish or preparation of the manuscript.

## Conflicts of Interest

The authors declare no conflicts of interest.

## Supporting information


**Figure S1:** Comparison of model error rates (MAE) for predicting three key soil functions based on data aggregation type. The performances of four distinct machine learning algorithms, Histogram Gradient Boosting Regression (HGBR), Random Forest Regression (RFR), Support Vector Regression (SVR) and Extreme Gradient Boosting Regression (XGBoost), were compared for the prediction of three separate soil functions: Soil organic carbon (SOC) stock (Panel A), Mean weight diameter (MWD) (Panel B) and Macroporosity (Panel C). Model performance is expressed as the Mean Absolute Error (MAE) between the predicted and observed values (y‐axis), quantifying the specific prediction error rates in the original units of the soil functions. Boxplots display the distribution of MAE values for each algorithm, evaluated on the independent testing sets across 10 random train/test split iterations, under four input omics data aggregation strategies: Absolute abundances (green), Centered Log‐Ratio transformation (orange), Functional aggregation (blue) and Taxonomic aggregation (pink). Statistical significance of the interaction between the machine learning model and the aggregation strategy was assessed using Duncan's Multiple Range Test following a two‐way ANOVA, with iteration included as a blocking factor (α=0.05).
**Figure S2:** SHAP dependence plots for the 25 most important determinants of SOC stock. Each subplot illustrates the relationship between a specific feature's value (x‐axis) and its impact on the model prediction (y‐axis, SHAP value). Positive SHAP values indicate a contribution toward an increased predicted SOC stock, while negative values indicate a decrease.
**Figure S3:** SHAP dependence plots for the 25 most important determinants of MWD. Each subplot illustrates the relationship between a specific feature's value (x‐axis) and its impact on the model prediction (y‐axis, SHAP value). Positive SHAP values indicate a contribution toward an increased predicted MWD, while negative values indicate a decrease.
**Figure S4:** SHAP dependence plots for the 25 most important determinants of macroporosity. Each subplot illustrates the relationship between a specific feature's value (x‐axis) and its impact on the model prediction (y‐axis, SHAP value). Positive SHAP values indicate a contribution toward an increased predicted macroporosity, while negative values indicate a decrease.
**Table S1:** Hyperparameter search spaces and final optimal values for the evaluated machine learning algorithms. The search spaces were defined for Bayesian optimization using Hyperopt. The final optimal hyperparameters retained for predicting soil organic carbon (SOC) stock, mean weight diameter (MWD) and macroporosity (MPR) are detailed in the respective columns. Values represent the optimized configurations from the best‐performing cross‐validation iteration (highest R2) utilizing the absolute abundances (ABS) omics data aggregation strategy, which was specifically selected to conduct the SHAP interpretability analysis.

## Data Availability

The raw sequencing data generated in this study have been deposited in the NCBI Sequence Read Archive (SRA) under BioProject PRJNA1210483. Benefit‐Sharing Statement: Benefits from this research accrue from the sharing of our data and results on public databases as described above.
