# Supplementary material for: An Interpretable Machine Learning Approach to Ecologically Characterize Soil Carbon and Structure From Multi‐Kingdom Microbiome, Texture and Climate
Source: Mol Ecol. 2026 Sep 1;35(17):e70535. doi: 10.1111/mec.70535 (PMC13531267; doi:10.1111/mec.70535)
Supplement: Supplementary file 1 [file MEC-35-e70535-s001.pdf]

Supplementary Material: An interpretable machine learning approach to ecologically characterize soil carbon and structure from multi-kingdom microbiome, texture, and climate

Thomas Jeanne

Appendix S1: Supplemental Figures

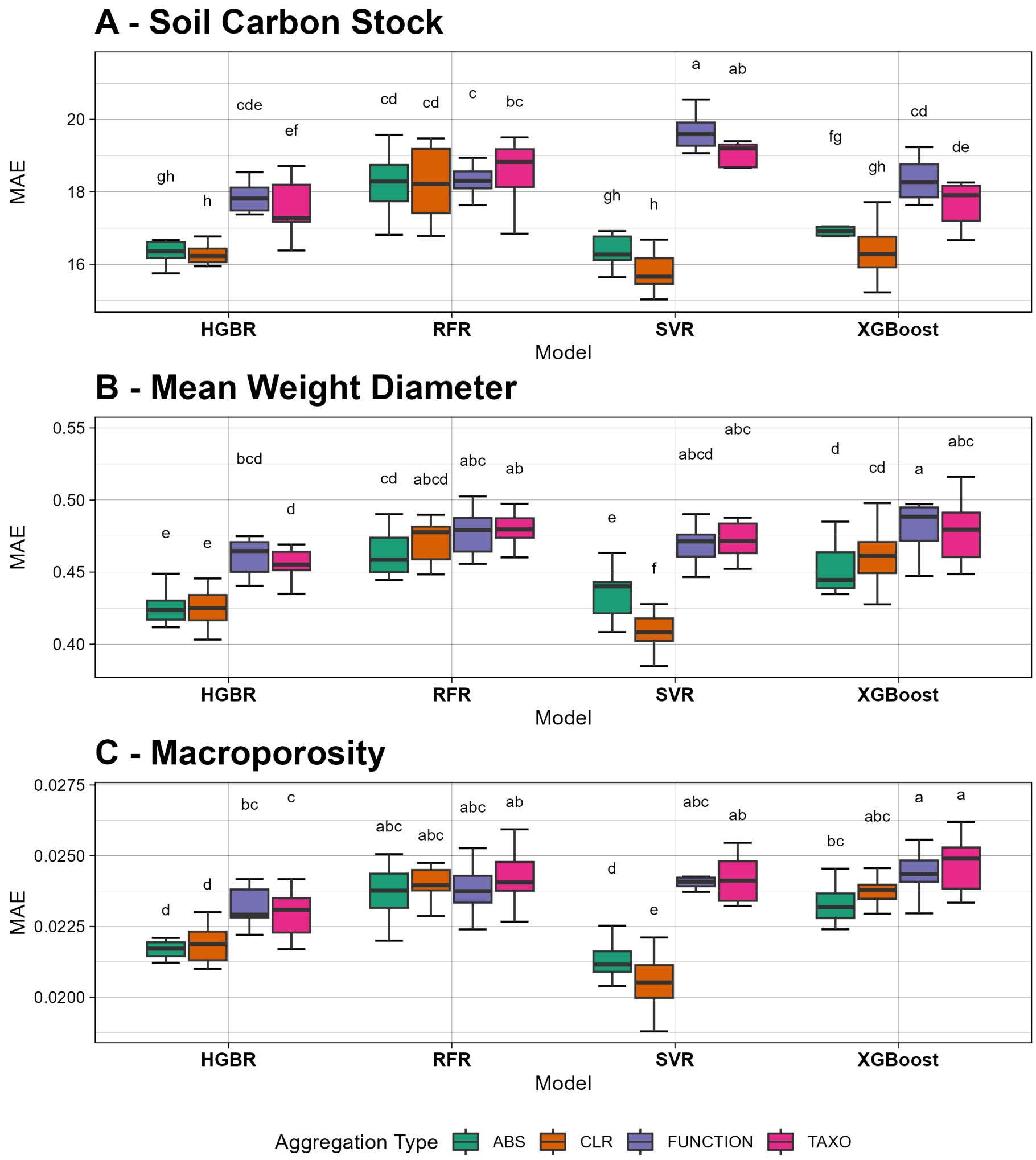

Figure S1: **Comparison of model error rates (MAE) for predicting three key soil functions based on data aggregation type.** The performances of four distinct machine learning algorithms, Histogram Gradient Boosting Regression (HGBR), Random Forest Regression (RFR), Support Vector Regression (SVR), and Extreme Gradient Boosting Regression (XGBoost), were compared for the prediction of three separate soil functions: Soil organic carbon (SOC) stock (Panel A), Mean weight diameter (MWD) (Panel B), and Macroporosity (Panel C). Model performance is expressed as the Mean Absolute Error (MAE) between the predicted and observed values (y-axis), quantifying the specific prediction error rates in the original units of the soil functions. Boxplots display the distribution of MAE values for each algorithm, evaluated on the independent testing sets across ten random train/test split iterations, under four input omics data aggregation strategies: Absolute abundances (green), Centered Log-Ratio transformation (orange), Functional aggregation (blue), and Taxonomic aggregation (pink). Statistical significance of the interaction between the machine learning model and the aggregation strategy was assessed using Duncan's Multiple Range Test following a two-way ANOVA, with iteration included as a blocking factor ($\alpha = 0.05$).

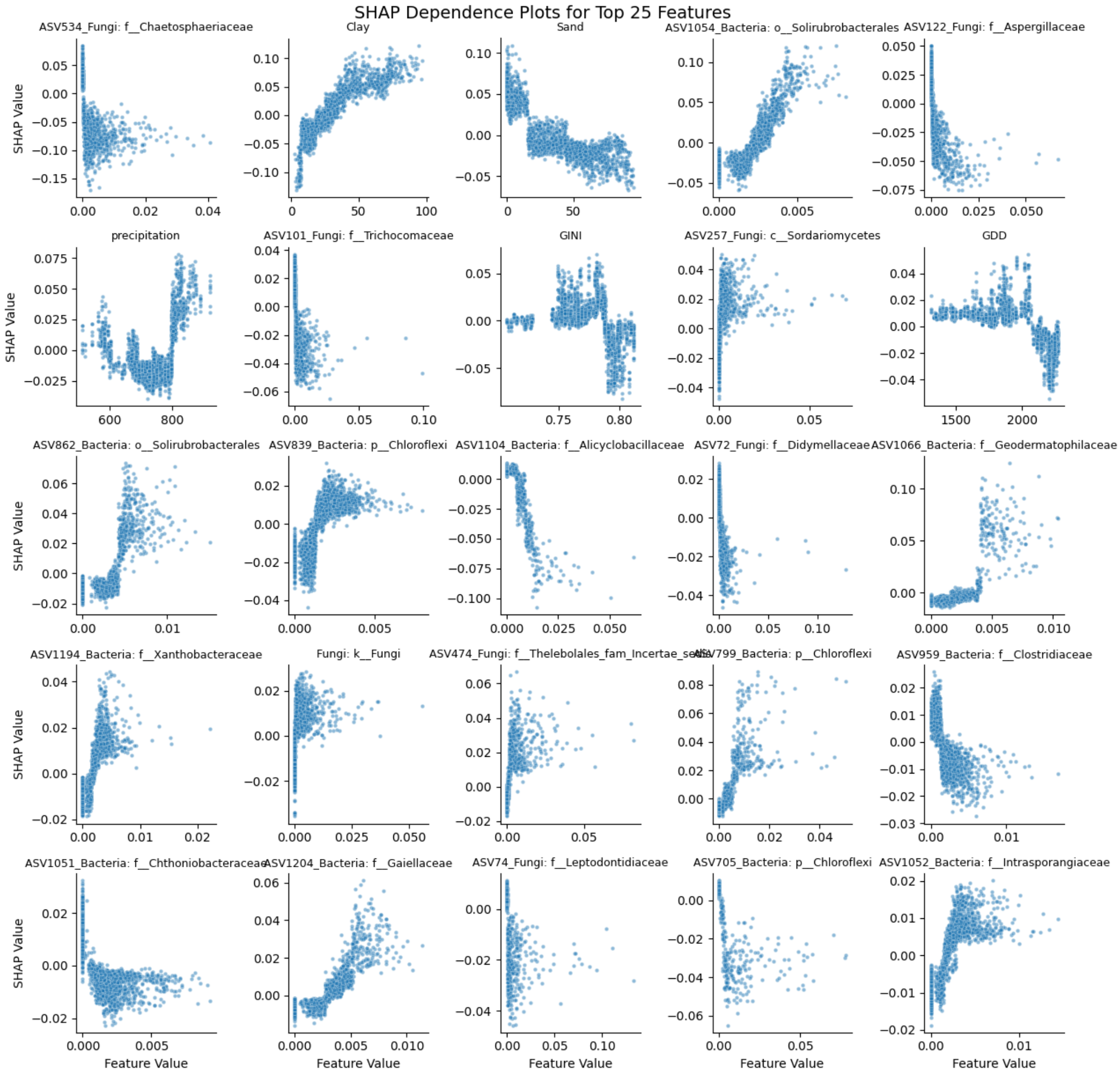

Figure S2: **SHAP dependence plots for the 25 most important determinants of SOC stock.** Each subplot illustrates the relationship between a specific feature's value (x-axis) and its impact on the model prediction (y-axis, SHAP value). Positive SHAP values indicate a contribution toward an increased predicted SOC stock, while negative values indicate a decrease.

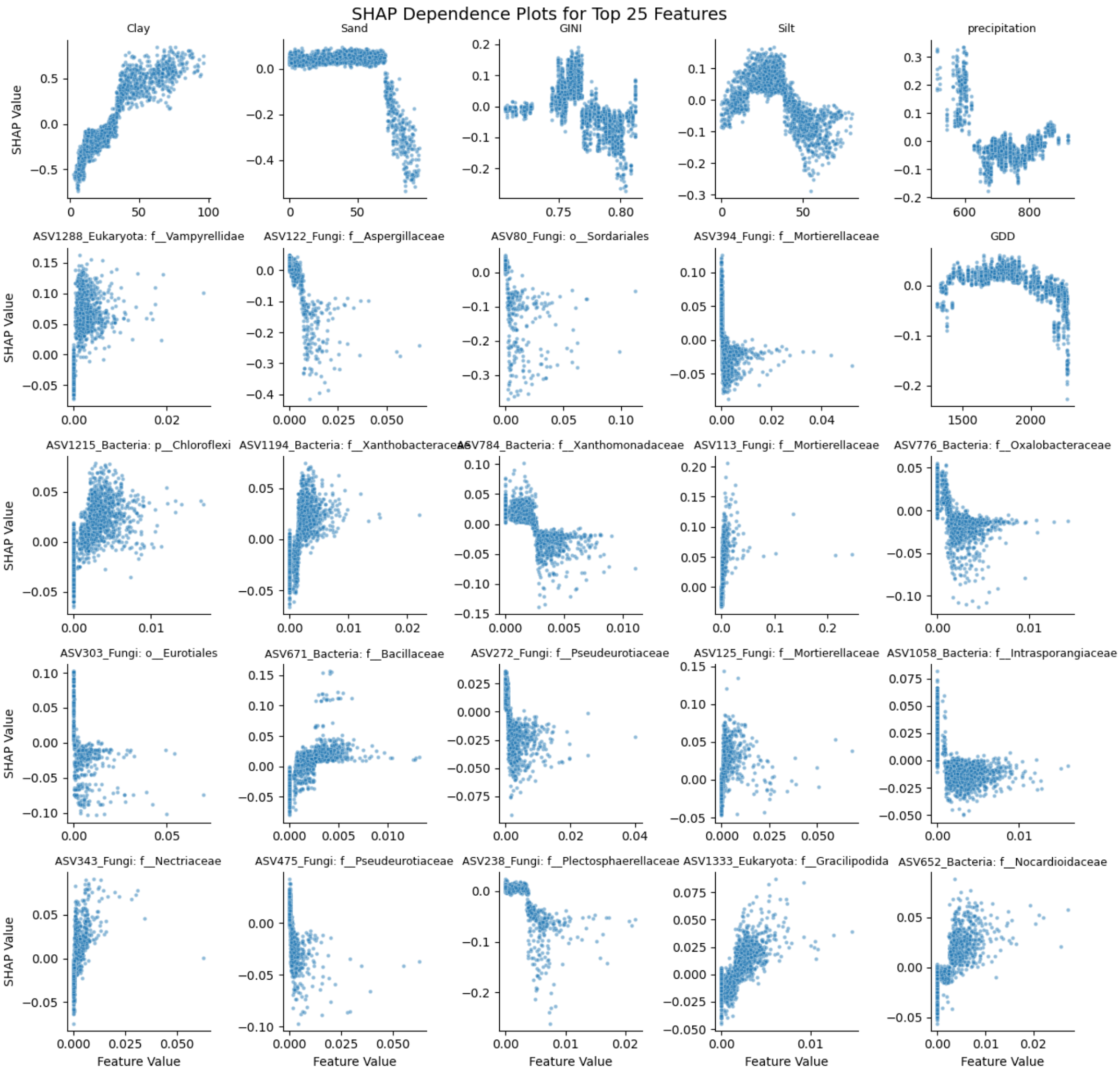

Figure S3: **SHAP dependence plots for the 25 most important determinants of MWD.** Each subplot illustrates the relationship between a specific feature's value (x-axis) and its impact on the model prediction (y-axis, SHAP value). Positive SHAP values indicate a contribution toward an increased predicted MWD, while negative values indicate a decrease.

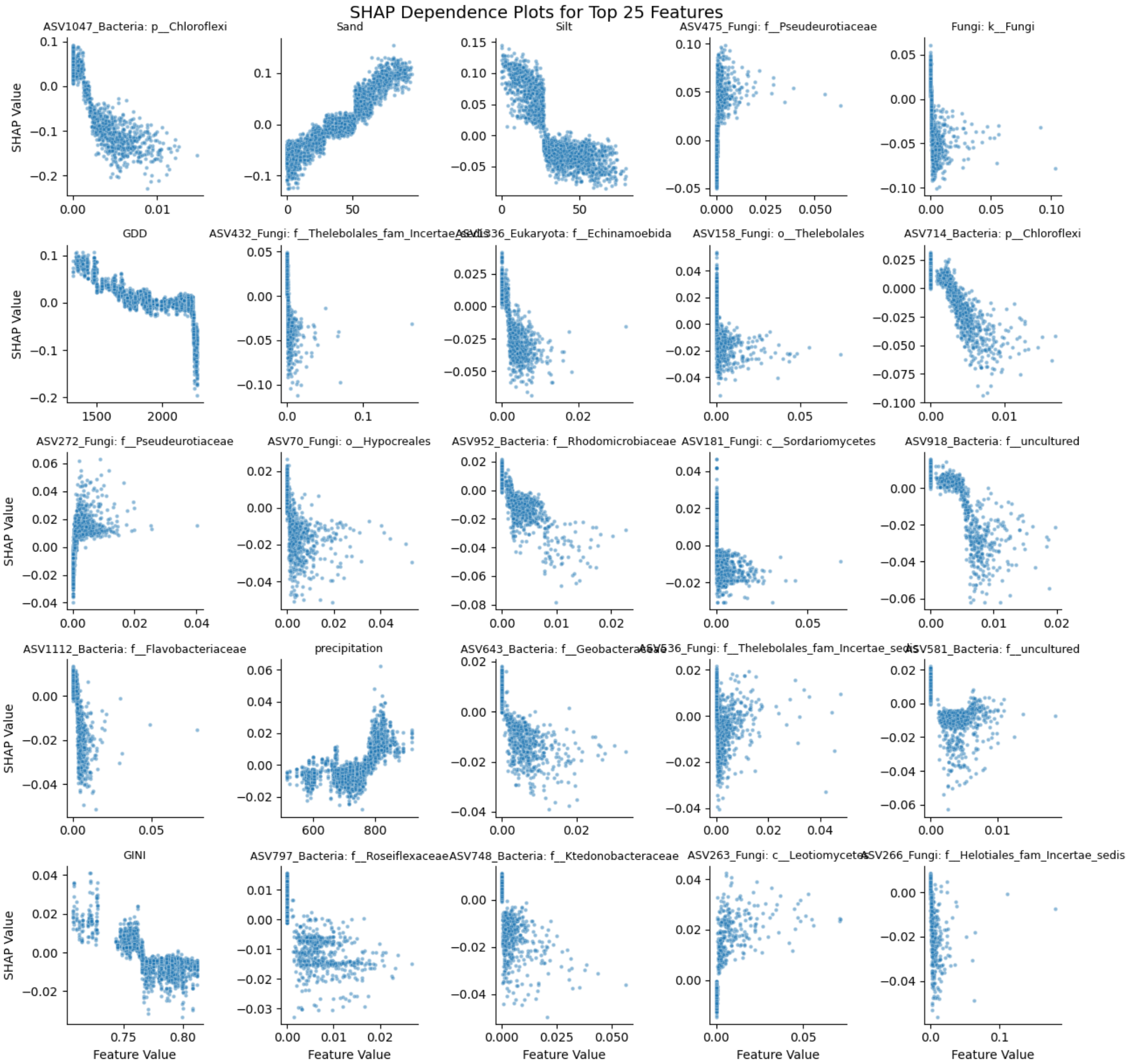

Figure S4: **SHAP dependence plots for the 25 most important determinants of macroporosity.** Each subplot illustrates the relationship between a specific feature's value (x-axis) and its impact on the model prediction (y-axis, SHAP value). Positive SHAP values indicate a contribution toward an increased predicted macroporosity, while negative values indicate a decrease.

Table S1: **Hyperparameter search spaces and final optimal values for the evaluated machine learning algorithms.** The search spaces were defined for Bayesian optimization using Hyperopt. The final optimal hyperparameters retained for predicting soil organic carbon (SOC) stock, mean weight diameter (MWD), and macroporosity (MPR) are detailed in the respective columns. Values represent the optimized configurations from the best-performing cross-validation iteration (highest R^2) utilizing the absolute abundances (ABS) omics data aggregation strategy, which was specifically selected to conduct the SHAP interpretability analysis.

Algorithm	Hyperparameter	Search Space (Distribution / Range)	Opt. SOC	Opt. MWD	Opt. MPR
**Random Forest (RFR)**	n_estimators	Discrete Uniform [100, 1500], step: 10	680	340	1200
	max_features	Choice: [1.0, 'sqrt']	1.0	1.0	1.0
	max_depth	Discrete Uniform [10, 300], step: 10	120	170	180
	min_samples_split	Discrete Uniform [2, 15], step: 1	3	3	6
	min_samples_leaf	Discrete Uniform [1, 5], step: 1	1	1	1
	bootstrap	Choice: [True, False]	True	True	True
	criterion	Choice: ['squared_error', 'absolute_error', 'friedman_mse', 'poisson']	friedman_mse	poisson	poisson
**HGBR**	learning_rate	Uniform [1e-6, 0.5]	0.1487	0.1151	0.1071
	loss	Choice: ['absolute_error', 'gamma', 'squared_error', 'poisson']	poisson	squared_error	poisson
	max_depth	Choice: [None, Discrete Uniform [1, 200], step: 1]	None	129	None
	max_features	Uniform [0.01, 1.0]	0.7878	0.8621	0.2944
	tol	Uniform [1e-10, 1e-3]	5.14×10^{-4}	2.74×10^{-4}	7.94×10^{-4}
	min_samples_leaf	Discrete Uniform [1, 75], step: 1	22	18	16
**XGBoost**	objective	Choice: ['reg:squarederror', 'reg:squaredlogerror', 'reg:absoluteerror']	reg:squarederror	reg:squarederror	reg:absoluteerror
	eta (learning rate)	Uniform [0, 1.0]	0.1842	0.2938	0.1260
	gamma	Uniform [0, 5.0]	0.7035	0.3878	3.472
	max_depth	Discrete Uniform [0, 100], step: 1	40	7	82
	min_child_weight	Discrete Uniform [0, 100], step: 1	0	12	34
	max_delta_step	Discrete Uniform [0, 100], step: 1	18	7	51
	subsample	Uniform [0.1, 1.0]	0.7633	0.9188	0.7706
	lambda	Uniform [0, 100]	86.67	14.29	27.00
	alpha	Uniform [0, 100]	93.76	4.948	16.56
	max_leaves	Discrete Uniform [0, 100], step: 1	69	14	0
**SVR**	C	Uniform [0.0, 20.0]	5.335	2.020	16.27
	epsilon	Log-Uniform [1e-10, 1e-2]	2.34×10^{-5}	3.40×10^{-8}	9.57×10^{-7}
	coef0	Uniform [0, 10.0]	1.357	0.028	0.188
	tol	Log-Uniform [1e-10, 1e-1]	1.55×10^{-4}	3.21×10^{-6}	2.46×10^{-3}
	kernel	Choice: ['rbf', 'sigmoid', 'poly' (degree: Discrete Uniform [0, 5])]	poly (deg.=4)	poly (deg.=2)	poly (deg.=2)
	gamma	Choice: ['auto', 'scale']	auto	auto	auto
